# Quality of Reporting in Preclinical Urethral Tissue Engineering Studies: A Systematic Review to Assess Adherence to the ARRIVE Guidelines

**DOI:** 10.3390/ani11082456

**Published:** 2021-08-21

**Authors:** Tariq O. Abbas, Abubakr Elawad, Abdul Kareem Pullattayil S., Cristian Pablo Pennisi

**Affiliations:** 1 Regenerative Medicine Research Group, Department of Health Science and Technology, Aalborg University, 9220 Aalborg, Denmark; cpennisi@hst.aau.dk; 2 Pediatric Urology Section, Sidra Medicine, Doha 26999, Qatar; aelawad@sidra.org; 3 College of Medicine, Qatar University, Doha 2713, Qatar; 4 Weill Cornell Medicine Qatar, Doha 24144, Qatar; 5 Clinical Library, Sidra Medicine, Doha 26999, Qatar; apullattayil@gmail.com

**Keywords:** urethral strictures, hypospadias, animal experiments, quality assessment, translational research

## Abstract

**Simple Summary:**

We have conducted a systematic review to investigate the quality of reporting in preclinical experiments exploring tissue engineering approaches for urethral repair. This was performed based on the Animal Research: Reporting of In Vivo Experiments (ARRIVE) guidelines in a total of 28 articles from 2014 to 2020. Inadequate reporting of the essential points of research experiments was observed that could remarkably affect clarity, reproducibility, and translatability. A complete statement of the ethical review permission and guidelines followed was missing in 54% of the studies. Details to ensure reproducibility of the studies, such as animal housing, husbandry, and anesthetics, were infrequently reported. No paper stated the sample size estimation methodology. The quality of reporting improved marginally over the study period. We encourage the utilization of the ARRIVE checklist items when reporting preclinical studies to help the publication of manuscripts that would allow a precise judgment of their scientific merit.

**Abstract:**

Preclinical research within the area of urethral tissue engineering has not yet been successfully translated into an efficient therapeutic option for patients. This gap could be attributed, in part, to inadequate design and reporting of the studies employing laboratory animals. In this study, a systematic review was conducted to investigate the quality of reporting in preclinical studies utilizing tissue engineering approaches for urethral repair. The scope was on studies performed in rabbits, published between January 2014 and March 2020. Quality assessment of the data was conducted according to the Animal Research: Reporting of in Vivo Experiments (ARRIVE) guidelines by the scoring of a 38-item checklist in different categories. A total of 28 articles that fulfilled the eligibility criteria were included in the study. The range of ARRIVE score was from 0 to 100, taking into consideration having reported the item in question or not. The mean checklist score was 53%. The items that attained the highest scores included the number of animals utilized, the size of control and experimental groups, and the definition of experimental outcomes. The least frequently reported items included the data regarding the experimental procedure, housing and husbandry, determination and justification of the number of animals, and reporting of adverse events. Surprisingly, full disclosure about ethical guidelines and animal protocol approval was missing in 54% of the studies. No paper stated the sample size estimation. Overall, our study found that a large number of studies display inadequate reporting of fundamental information and that the quality of reporting improved marginally over the study period. We encourage a comprehensive implementation of the ARRIVE guidelines in animal studies exploring tissue engineering for urethral repair, not only to facilitate effective translation of preclinical research findings into clinical therapies, but also to ensure compliance with ethical principles and to minimize unnecessary animal studies.

## 1. Introduction

Urethral repair is considered a complex task for urologists, where the greatest demanding clinical needs occur following urethral strictures in adults and congenital anomalies (e.g., hypospadias) in children. In adults, it has been estimated that 0.1% of men above the age of 65 years suffer from urethral strictures, which can occur secondary to different etiologies, including pelvic trauma, lichen sclerosus, non-specific urethritis, and iatrogenic injuries [1]. The selection of the surgical management approach is based on the extent and etiology of the stricture. For distal strictures, the approach includes initial endoscopic urethrotomy or dilatation, and subsequent surgical interventions are usually reserved to when the urethrotomy fails, or the stricture recurs. For short strictures, anastomotic urethroplasty generally solves the problem. The preferred surgical approach in strictures larger than 1 cm or complicated cases is replacement urethroplasty using an autologous graft that can be used either as a patch or in full circumference. Although different sources of grafting material have been tried, buccal mucosa is currently considered the preferred option owing to its inherent lack of hair, compatibility with a moist environment, and a low rate of donor site morbidity [2]. In pediatric patients, hypospadias has an incidence of around 1 per 300 male newborns, where the shortage of the urethra is a surgical challenge [3]. The currently practiced surgical techniques for the management of these diseases have high complication rates and need specific skills to be applied optimally [3,4,5,6,7]. For both adult and pediatric patients, there is a wide consensus about the need for further consolidated basic research, including the use of tissue engineering and regenerative medicine techniques for urethral reconstruction [8,9,10,11].

Among the various animal models used to investigate male urethral repair strategies, the rabbit model has been by far the most popular choice [10,12,13]. The male rabbit’s urethra is easily accessible and possesses remarkable histological and functional similarities to the human urethra, such as a thin epithelial layer supported by the highly vascularized *spongiosum* and a urethral smooth muscle layer contributing to the urethral tone [14,15,16,17,18]. In addition, the size of an adult rabbit’s urethra is comparable to that of a male infant, allowing the use of transurethral instrumentation and procedures employed in pediatric surgery. Studies using rabbits have, therefore, been instrumental in demonstrating the feasibility of urethral reconstruction using a variety of synthetic and natural polymeric matrices [8,9,10]. Several approaches have been under scrutiny, most of which have shown the ability to support the recovery of normal urethral architecture and function, with a similar performance to that of autologous tissue grafts. However, a recent meta-analysis of 63 preclinical and 13 human studies of tissue engineering for urethral reconstruction revealed that the efficacy of these approaches could not be defined because of the lack of well-controlled preclinical investigations. The study also revealed that the promising preclinical results obtained using cell-laden matrices could surprisingly not be translated into the clinical studies [10]. 

Studies in other fields have also shown a number of difficulties in assessing the efficacy or translating the results from animal research to the clinical context. The issues include physiological variations among species and strains [19], lack of randomization and blinding [20], inadequate reporting of methods and materials, and the publication bias of not describing trials with adverse or indeterminate outcomes, which derivate to an overestimation of the impact of a therapy [21]. In 2009, the National Centre for the Replacement, Refinement, and Reduction of Animals in Research (NC3Rs) examined the nature of the reporting, experimental design, and statistical analysis in 271 published preclinical experiments. The survey showed several shortcomings in study design, statistical analysis, and reporting, and inspired the publication of the Animal Research: Reporting In Vivo Experiments (ARRIVE) guidelines in 2010 [22,23,24]. The checklist consists of 20 items that cover the critical data to be reported in a preclinical scientific paper. Despite increased awareness in the scientific community, however, which includes the adoption of the guidelines by more than 1000 scientific journals, the quality of reporting has not significantly improved in various research fields [25,26,27]. In 2018, the NC3Rs formed an international working group involving journal editors, researchers, and statisticians from a variety of fields with the aim of reviewing and updating the guidelines [28]. As a result, a revised version of the guidelines has recently been published (ARRIVE 2.0) [29].

While the reasons the positive results obtained in preclinical studies for urethral repair have not been reproduced in the subsequent clinical trials are complex, poorly designed experiments and a lack of quality in reporting might be some of the main reasons that hinder clinical translation [30]. However, the quality of reporting in preclinical urethral tissue engineering studies remains unclear. Moreover, it is unknown if the quality of reporting has improved as a consequence of the introduction of the ARRIVE guidelines. This systematic review aims to address these research questions by performing a quality assessment using the ARRIVE guidelines as a checklist. The scope of the review was restricted to studies using rabbits, which are the most employed animal model for preclinical research in urethral reconstruction. The outcome assessment of different tissue engineering approaches is beyond the scope of this review, as has been addressed in other systematic reviews in the literature [12,31,32].

## 2. Methodology

### 2.1. Literature Search

**Figure 1:**
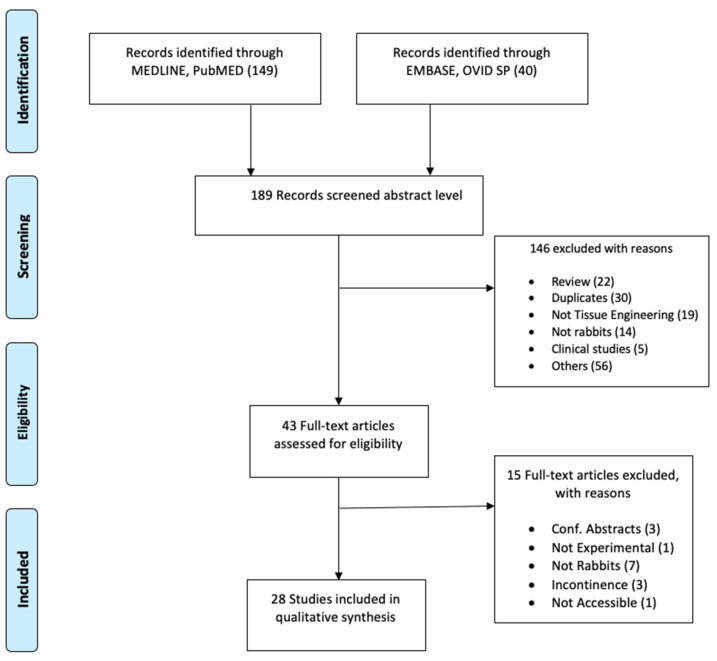
PRISMA flow chart to outline the inclusion process for the articles in this study.

Two separate searches were conducted in the databases MEDLINE of PubMed and EMBASE of OVID SP in March 2020. The search terms selected were as follows: rabbit, tissue engineering, stem cell, scaffolds, autologous graft, urethral graft, urethral reconstruction, regenerative medicine, reconstructive surgery, urethra, and animal experimentation. The search fields were controlled by database fields such as MeSH term, Text Word, and All Fields appropriate to the databases. “Publication date: 01/01/2014 to present” and “English language” filters were used. As the ARRIVE guidelines were first released in 2010, we selected to start our search in 2014, assuming that four years would allow the authors of preclinical studies sufficient time to plan, perform, and publish the results according to the guidelines. Details of the search are represented in the PRISMA flow diagram (Figure 1).

### 2.2. Screening

All retrieved publications were screened at the abstract level initially by the authors T.A. and A.K.P.S. The inclusion criteria were studies that evaluated urethral tissue engineering techniques in rabbits. Exclusion criteria included reviews, studies concerning reconstruction of other parts of the urinary tract, clinical studies, duplicates, and studies using other animal models (flow chart diagram, Figure 1). Group discussions resolved disputes regarding the appropriateness of an article. Eligible articles were included for full-text analysis.

### 2.3. Data Extraction

Extraction into a standardized data framework derived from the ARRIVE checklist [22] was conducted by two independent reviewers (A.K.P.S. and A.A.). The ARRIVE guideline consists of 20 items, some of which are further divided in subitems. For the purpose of this work, a list of 38 items was elaborated, which includes the items and subitems from the original ARRIVE guideline (Supplementary Table S1). Each of these 38 items was evaluated as “Yes” or “No” to indicate whether it was reported in the study or not. For some of the questions, a third option, “Not Applicable (N/A)”, was included to indicate items that were not relevant for the study (for example, in experiments employing only one experimental group, item 11a concerning allocation becomes N/A). Specific operational instructions (Supplementary Table S1) were provided to both reviewers before they read the selected full-text articles and extracted the data blinded to the analysis from the other reviewer. A training phase through the detailed description and examples of scoring was conducted among the authors before the commencement of the data extraction. Inconsistent data were consequently settled by an additional independent researcher (T.A.).

### 2.4. Data Analysis

The data were compiled employing a Microsoft Excel spreadsheet and analyzed using IBM SPSS Statistics (version 21). For each of the selected studies, a score was calculated, which represents the percentage of positively reported items. The score was calculated using the following formula:

1 $$Score=\left(\frac{Nyes}{38-Nna}\right)\times 100$$

where *Nyes* = number of “Yes” entries, *Nna* = number of “Not Applicable” entries, and 38 is the total number of items in the ARRIVE guideline. The units of analysis were the individual articles when assessing the scores, and the single ARRIVE item when assessing their adherence across studies. A further analysis was performed to assess the adherence of the studies to several subitems within the ARRIVE checklist. A Mann–Kendall nonparametric test was used to assess whether the scores had a monotonic trend over the years. This is a simple, but robust non-parametric test that does not require the data to be normally distributed or follow a linear trend. The intraclass correlation coefficient (ICC) analysis was utilized to examine the inter-rater agreement between the two reviewers. The ICC was selected because it reflects both degree of correlation and agreement between measurements. ICC values <0.5 are indicative of poor reliability, 0.5–0.75 indicate moderate reliability, 0.75–0.9 indicate good reliability, and >0.90 indicate excellent reliability [33]. Statistical significance was set at *p* < 0.05.

## 3. Results

As shown in Figure 1, a total of 189 articles were initially screened after the literature search. Following the inclusion and exclusion criteria, a total of 43 studies were selected for full-text reading (Figure 1). Only 28 studies were considered eligible for quality appraisal [34,35,36,37,38,39,40,41,42,43,44,45,46,47,48,49,50,51,52,53,54,55,56,57,58,59,60,61]. These studies comprise a range of approaches and scaffolds for urethral repair in rabbits, which are summarized in the Supplementary Table S2. The table provides details about the strain, sex, age, weight, number of animals, graft approach, material, and duration of the implantation. The numbers of rabbits in each experiment was 20 on average and ranged between 7 and 36, and the post-implantation follow-up duration was 5 months on average and varied between two weeks and nine months. All studies used male rabbits, except one study that included male and female animals. The most commonly studied approach was using acellular matrices as a patch (21 studies; length average (15 mm) range (5–20 mm)) versus tubes (7 studies; length average (20 mm) range (10–30 mm)).

**Figure 2:**
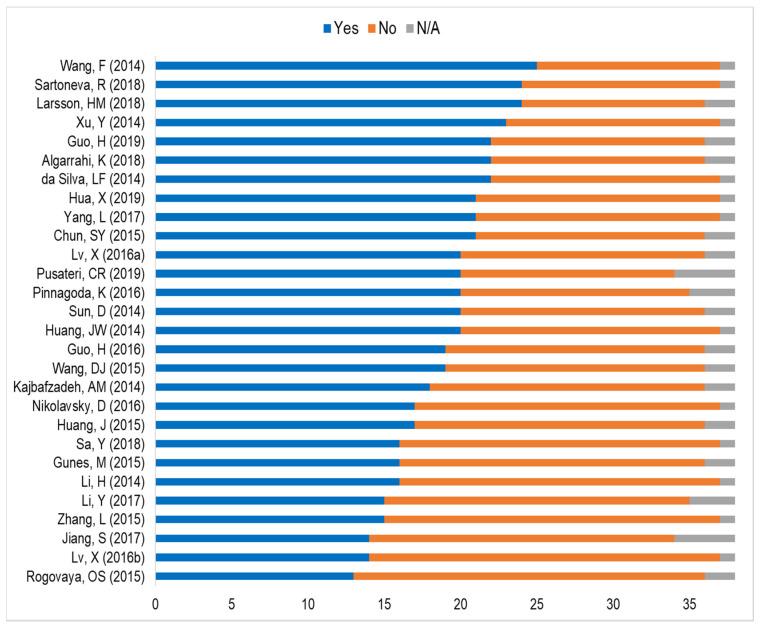
Bar chart displaying the frequencies of the options ‘Yes’, ‘No’, and ‘N/A’ among the 28 articles selected for the analysis.

After the full analysis was completed by the reviewers, the data were compiled in a spreadsheet (Supplementary File S1), which includes the responses to the 38 items in the operational table based on the ARRIVE guidelines (Supplementary Table S1). The ICC analysis to examine the inter-rater agreement had a value of 0.84 (95% CI 0.802–0.943), which is considered good. The frequencies of the options “Yes”, “No”, and “N/A” following assessment of the selected studies are depicted in Figure 2. It is evident that none of the analyzed articles scored “Yes” for all the items in the checklist or fully complied with the ARRIVE guidelines.

**Figure 3:**
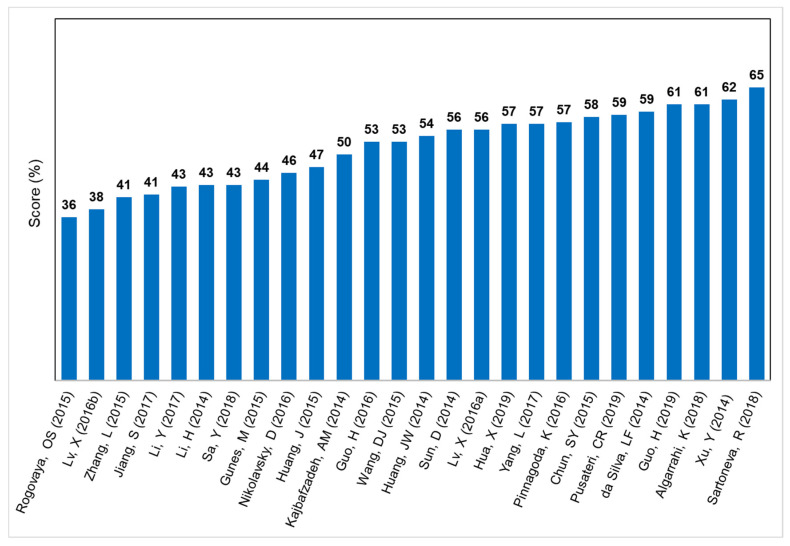
Bar chart displaying the scores of the analyzed articles. Scores represent the percentage adherence to the ARRIVE guidelines on a scale from 0 to 100.

The scores of each individual study, which are based upon the information presented in Figure 2 and compiled in the Supplementary File S1, are presented in Figure 3.

**Figure 4:**
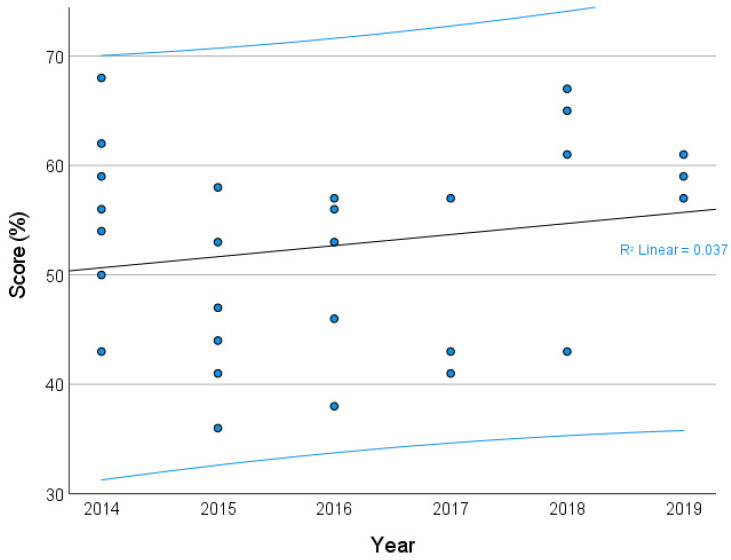
Trend in the score from 2014 to 2020. The trendline presented is from the Mann–Kendall trend analysis on the median of the scores. The stapled lines indicate the 95% confidence interval.

The bar graph displays all the scores assigned to each of the 28 studies, from the lowest (36%) to the highest (65%). The average score was 53%. The general trend of the ARRIVE scores of the included articles over the 5-year study period is shown in Figure 4. The Kendall’s rank correlation coefficient (Tau) was found to be very low (0.102, *p* = 0.479), indicating a weak upward trend in the score values over the analyzed period (2014–2020). 

**Figure 5:**
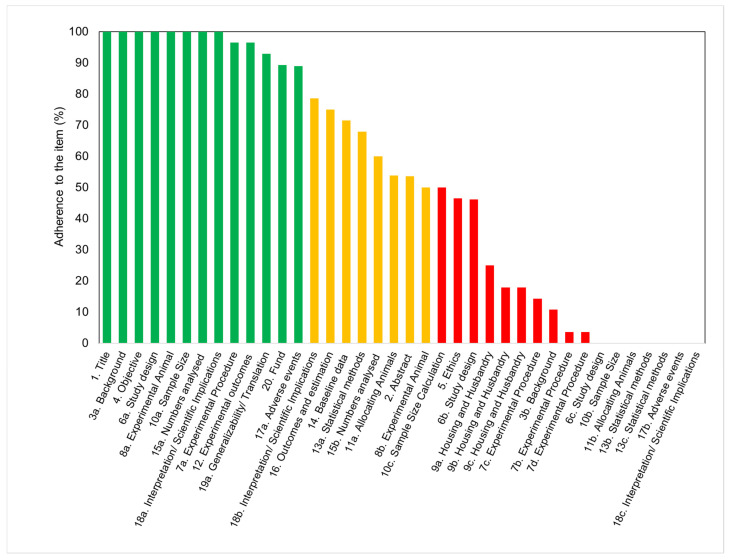
Bar chart showing the degree of adherence (in %) to the 38 items in the ARRIVE checklist. The data were clustered to display the items in which studies had a high degree of adherence (between 80 and 100%, in green), medium degree of adherence (between 50 and 79%, in yellow), and low degree of adherence (0 to 49%, in red). Items are indicated by their corresponding numbers and a label associated with their content.

The scores for each of the checklist items are shown in Figure 5. The data were clustered into three groups to evidence the level of adherence to the guidelines of each of the 38 items in the checklist. While 34% of the items (13/38) appeared under the green category (agreement between 80 and 100%), 21% of the studies (8/38) appeared in the orange category (agreement between 50 and 79%), and 45% of the items (17/38) were associated with the red category (agreement between 0 and 49%).

The items that attained the highest scores included the number of animals utilized, the size of experimental and control groups, and the definition of experimental outcomes. A description of the study background, including context and rationale, was also adequately provided in all of the studies. However, variables relevant to the reproducibility of the experiments were not often disclosed. The least frequently reported checklist items (only found in ≤10% of the studies) were items 18c (interpretation), 7b (experimental procedures), 17b (adverse events), 6c (experimental unit), 13b (analysis unit), 7d (procedure rational), 11b (animal allocation), 13c (statistical design), and 10b (sample size). These items are related to information on test methods, sample size calculation, statistical approaches, adverse events, and interpretation/scientific outcomes. Although statistical methods were disclosed and described in almost two-thirds of the articles, the statement of statistical methodology was frequently inadequate. Most articles did not include information about data distribution, definition of the unit of analysis, or justification for choosing a specific analytical method. Surprisingly, none of the articles disclosed the approach to verify that the assumptions for the statistical methods were met. Although every article described the experimental results, some of them failed in specifying the primary and secondary outcomes.

The adherence of the studies to the different parts, rather than the overall item, is shown separately in a table format (Table 1). Concerning the compliance to ethical standards, 92% of the analyzed studies stated that the protocol was approved and 89% referred to national or international guidelines (Table 1a). However, a complete ethical statement, disclosing both the approval of the protocol and guidelines followed, was only present in 46% of the studies. Concerning study design, there were various essential items that were poorly reported. While recording of randomization (6.b) scored 46%, none of the 28 studies reported sample size estimation or steps to reduce assessment bias (Table 1b). Regarding item 7a (experimental procedure), while it scored 96% owing to the majority of studies reporting surgical procedures and anesthesia, very few studies reported post-operative analgesia or euthanasia (Table 1c). Regarding the details of the animals, the most frequently reported information was the weight. However, only 14% of the studies provided the age of the animals (Table 1d). Details concerning the animal’s housing (9a) were infrequently listed (less than 30% of all studies). Only few studies reported the type of facility, cage, bedding material, or number of cage companions (Table 1e). Data about nutritional aspects and environment, such as temperature, humidity, and access to water and food, were also infrequently reported. No environmental enrichment was reported in any of the studies (Table 1f). In relation to the study limitations (18.b), most studies stated general limitations and potential sources of bias. However, only few articles addressed the limitations of the animal model or imprecision of the results (Table 1g).

## 4. Discussion

Studies utilizing animal models play a key role in scientific discovery, given that the tests are composed, executed, evaluated, and appropriately communicated following internationally accepted guidelines. A loss of transparency in preclinical research studies has been recognized [62]. Several fundamental components of the experimental design are often overlooked in these published studies, which contributes to irreproducibility of the experiments. Preclinical studies investigating tissue engineering for urethral repair do not seem to be an exception.

Our study determined that the quality of reporting of some key items that influence the interpretation of the study was generally poor in preclinical studies of tissue engineering for urethral repair, and that the quality of reporting improved very slightly over the study period. Noticeably, some details to ensure the reproducibility of preclinical studies, such as animal housing, husbandry, and anesthetics, were infrequently reported, which may significantly affect the study results. For instance, studies have shown that the type of anesthetics may affect the long-term behavior of the animals [63]. Prager et al. stated that housing and husbandry conditions might significantly affect the behavior of rodents and, consequently, influence the results [64]. Confining animals to small cages or keeping several animals per cage after urethroplasty carries a potential risk of higher infection rates [65], and increases the chances of stents’ dislodgement [66] as well as surgical site trauma. There was a low degree of adherence to guidelines in items concerning experimental design, e.g., allocation of the animals to groups and assessor blinding. As studies have shown, these items are fundamental not only to ensure adequate statistical analysis, but also to comply with the ethical principles, as they intend to decrease subjective bias and minimize the number of animals utilized in the study [62,67]. Sample size calculation, randomization, and blinding were also considered to be crucial for transparency and validation of experiments as per the Landis core reporting standards [62]. Our results agree with data from other biomedical areas in which preclinical research has been carried out. For instance, a study on preclinical research done in the field of rheumatology revealed a lack of reporting of several important items, including the calculation of animal numbers (0%); allocation (0%); randomization (0%); housing and husbandry conditions (5%); and reflection on the replacement, reduction, and refinement principles (3Rs) (0%) [27]. Thus, while a lack of information does not appear to be unique to the field of urethral repair, it is important to stress that proper randomization, allocation, and blinding will be more likely to produce reliable results, which minimize the risk of bias and exaggerated effect sizes [68]. We have found that only three of the articles enrolled in our analysis discussed the “how and why” concerning the animal model utilized and its relevance to human pathology. We believe that this information is of significant importance in tissue engineering for urethral repair experiments, given the fact that no single animal model is considered the best representative for human pathology and significant differences exist between the human and animal genital anatomy.

Surprisingly, while most articles presented the essential features of the experimental animals (e.g., strain, sex, age, and weight), no study disclosed the sample size estimation method. While calculation of an accurate sample size might be quite challenging owing to difficulties in the estimation of complication rates and the inherent dispersion of the model, there are some valid alternative approaches to estimate the number of animals needed in an experiment. These approaches include, for instance, performing pilot experiments at a scale that would allow indicating the standard deviation or using Mead’s resource equation when power analysis is not possible. The resource equation approach is useful when there is no available information about standard deviation and/or it is challenging specifying an effect size likely to be clinically meaningful [67].

Another critical finding of our study was related to the lack of discussion about study limitations in the analyzed articles. Moreover, elaboration on the feasibility of translation of the findings into other animal species and human clinical trials was lacking in most of the discussion sections. Efforts should be made to empower the 3Rs principles, making sure to wisely utilize laboratory animals and the available alternative approaches. In addition, to ensure reliable translation of the findings in clinical trials, studies should include a statement indicating measures taken to reduce significant adverse events, as there is a reasonable chance that adverse events may occur as a result of the research procedures.

Our study did not assess whether or not the journals in the analyzed dataset supported the ARRIVE guidelines at the time of submission. While it was not possible to determine how the data might have been biased by publishers, there are several reasons that indicate that their influence has been modest. A recent study demonstrated that a change in the editorial policy of the Nature Publishing group, which requires authors to follow a set of strict guidelines for reporting in vivo research, did not substantially increase the overall compliance [69]. On the other hand, checklist assessments and completion by peer review were not practical, possibly owing to the busy nature of the review process [70]. Various outcomes have been reported in studies evaluating the impact that adherence to the ARRIVE checklist had on the quality of the articles published [71,72]. Interestingly, a randomized controlled trial revealed that mandating the completion of an ARRIVE checklist during paper submission did not help to promote adherence to the guidelines in the articles [73]. As studies have shown, current measures such as endorsement by reviewers and editors to comply with the ARRIVE checklist did not appear to significantly improve the quality of reporting [71,73]. Alternatively, the recently published PREPARE (Planning Research and Experimental Procedures on Animals: Recommendations for Excellence) guidelines [74] might be helpful to enforce more attention to experimental rigor at the earlier phases of the research process.

It is worth noting that the reporting checklists are not strictly universal for experiments related to the different types of interventional studies. Therefore, it is critically important to utilize a suitable type of reporting guidelines accordingly. The EQUATOR (enhancing the quality and transparency of health research) international network [75] enhances the adherence to fulfilling the requirements of adequate reporting in a sensible, transparent, and reproducible manner.

In our assessment of the literature, we employed the ARRIVE guidelines available at the time of the data analysis (version 1.0), which comprise a total of 38 discrete evaluation items. As a means to gain more insight into the quality of the published studies, it has been proposed to arbitrarily operationalize the checklist in more evaluation items. For instance, in a review on preclinical experiments of acute lung injury, Avey et al. have chosen to operationalize the checklist into 109 discrete sub-items [76]. However, this approach might transform the analysis of a large number of articles in a tedious and time-consuming process. To overcome these difficulties, the working group in charge of the revision of the guidelines previously suggested to organize the parts in the ARRIVE checklist into tiers indicating various degrees of priority [28]. This approach would allow prioritizing some of the key items in the checklist to initially minimize the burden of analysis and provide measures that could be more easily followed by the scientific community. As a conclusion of the work done by the group and concurrently with the completion of this study, ARRIVE version 2.0 has been released [29]. The new version prioritizes the items, adds new information, and provides an accompanying explanation and elaboration (E&E) document to improve the understanding of the application and rationale of each item. The checklist items have been divided into “ARRIVE Essential 10” with the minimum requirements in the manuscripts and “Recommended set” that adds context to the study described. This prioritization scheme is expected to improve adherence to ARRIVE guidelines by paying attention to basic items followed by the gradual fulfillment of the recommended items. 

One of the strengths of our study is that it provides an assessment approach that could be utilized to evaluate the reporting quality in several pre-clinical research fields. To value the training effects, there were two independent reviewers who individually scored the different items, and a third reviewer who decided on the discrepant points. Subjectivity of the assessment by the several observers might be considered a limitation in our study. Nevertheless, the great inter-observer agreement confirmed that the analysts had a consistent assessment criterion. Our reviews were performed on studies published between 2014 and 2020, and restricted to experiments on rabbits. When analyzing systematic reviews in the topic of urethral tissue engineering that include studies prior to 2014 and experiments using other animal models, it becomes evident that rabbits represent by far the most frequently used model, comprising 31 studies performed until January 2017. That represents approximately 73% of the total of the studies, versus 24% of studies in dogs and 3% in rats [10,12,31,32]. It is therefore reasonable to assume that our sample (28 studies) would be representative of the general reporting quality in preclinical urethral tissue engineering research. However, further research is required to determine if the results presented here would have been significantly affected by including studies prior to 2014 or studies in other animal species. Although we analyzed the quality of the reporting of animal experiments, we cannot reflect on the overall nature of each research study. Therefore, it would be challenging to judge whether such investigations are flawed owing to a lack of reporting of methodological details. Lastly, one of the limitations of our operationalization table is that it involves several sub items and partial reporting could be considered as “Yes”. For this reason, we have added sub-item analysis to assess the adherence to each single sub item rather than the overall item.

## 5. Conclusions

Our study recognized that published animal experiments studying tissue engineering approaches for urethral repair display inadequate reporting of fundamental information. The quality of reporting improved only marginally over the study period. Inadequate reporting of the critical points of research experiments could remarkably affect the clarity, reproducibility, and translatability. We encourage the utilization of the ARRIVE checklist items when reporting preclinical studies to help the publication of manuscripts that would allow a precise judgment of their scientific merit. This has the potential to enhance both the translatability of the findings to humans and the fulfilment of the ethical requirements and further supports objective comparison between different studies.

## Figures and Tables

**Table 1:** Analysis of adherence of the studies to selected items in the checklist.

**(a) Item 5: Ethical statements**	**Ratio**	**%**
Refers to guidelines	25/28	89
Approved protocol	26/28	92
**(b) Item 6b: Steps to minimize subjective bias in the study design**		
Random allocation	13/28	46
Blind assessment	0/28	0
**(c) Item 7a: Information about experimental procedures**		
Surgical procedure	20/28	71
Anesthesia	22/28	79
Post-operative analgesia	4/28	14
Method of euthanasia	4/28	14
**(d) Item 8a: Details about animals in the experimental design**		
Sex	28/28	100
Weight and age	4/28	14
Weight but not age	18/28	64
Age but not weight	0/28	0
**(e) Item 9a: Information about housing conditions**		
Type of facility	5/28	18
Type of cage	3/28	11
Bedding material	1/28	4
Number of cage companions	1/28	4
**(f) Item 9b: Information about nutritional aspects and environment**		
Access to water / food	3/28	11
Breeding program	2/28	7
Temperature/humidity	2/28	7
Light/dark cycles	2/28	7
Environmental enrichment	0/28	0
**(g) Item 18b: Disclosure of limitations in the results´ interpretation**		
General limitations, including potential sources of bias	21/28	75
Limitations of the animal model	10/28	36
Imprecision	8/28	29

## Data Availability

The data analyzed in this study are available as supplementary material (Supplementary File S1).

## Supplementary Materials

The following are available online at https://www.mdpi.com/article/10.3390/ani11082456/s1, Table S1: Operationalized checklist for the assessment of the quality of reporting in urethral tissue engineering studies. Excerpts from the original ARRIVE guidelines were reformulated as operational questions in the table. The ARRIVE guidelines are published in an open access article distributed under the terms of the Creative Commons Attribution License (CC-BY) [22]. Table S2: Summary of the main aspects of the analyzed studies. File S1: Data collected and analyzed in this study.
