# Supplementary material for: Quality of Reporting in Preclinical Urethral Tissue Engineering Studies: A Systematic Review to Assess Adherence to the ARRIVE Guidelines
Source: Animals (Basel). 2021 Aug 21;11(8):2456. doi: 10.3390/ani11082456 (PMC8388767; doi:10.3390/ani11082456)
Supplement: Supplementary file 1 [file animals-11-02456-s001.zip › Table S1.pdf]

Supplementary Table S1: Operationalized ARRIVE checklist for the assessment of the quality of reporting in urethral tissue engineering studies. The terms in arrive checklist were reformulated as questions (ARRIVE guidelines were published in PLOS biology under CC-BY license).

No.	ARRIVE item	Operational question	Answer		
			Yes	No	N/A
1.	1. Title	Is the description of the content of the article reported in an accurate and concise manner?			
2.	2. Abstract	Is there a summary of the background **AND** research objectives **AND** details of the species or strain of animal used **AND** key methods **AND** principal findings **AND** conclusions of the study reported in the abstract?			
3.	3a. Background	Is there a sufficient scientific background to explain the approach and rationale of the study?			
4.	3b. Background	Is there any explanation on how and why the specific animal model has been selected **OR** the study's relevance to human biology?			
5.	4. Objective	Are the objectives of the study **OR** the hypothesis being reported?			
6.	5. Ethics	Is there any disclosure of the ethical permissions **AND** the guidelines followed for care and use of animals?			
7.	6a. Study design	Is the number of the experimental **AND** control groups mentioned?			

		(N/A= If no control group has been used)			
8.	6b. Study design	Is randomization **OR** blinding provided?<br><br>(N/A= If there is a single experimental group)			
9.	6c. Study design	Is there an experimental unit reported?			
10.	7a. Experimental Procedure	Was/were anesthesia **OR** analgesia **OR** surgical procedure **OR** method of euthanasia described?			
11.	7b. Experimental Procedure	Is the time (during the day) of the procedure reported?			
12.	7c. Experimental Procedure	Is the location of the study procedure provided?			
13.	7d. Experimental Procedure	Is the rationale for selecting a specific anesthetic **OR** surgical approach **OR** drug dose used provided?			
14.	8a. Experimental Animal	Is/are the sex **OR** age **OR** weight of the animals provided?			
15.	8b. Experimental Animal	Is there any further relevant information about the experimental animal model such as the source of animals **OR** international strain nomenclature **OR** genetic modification status (e.g. knock-out or transgenic) **OR** genotype **OR** health/immune status **OR** previous procedures mentioned?			
16.	9a. Housing and Husbandry	Are the details about type of facility (e.g. specific pathogen free) **OR** type of cage or housing **OR** bedding material **OR** number of cage companions reported?			

17.	9b. Housing and Husbandry	Are details about breeding program **OR** light/dark cycle **OR** temperature **OR** type of food **OR** access to food and water **OR** environmental enrichment provided?			
18.	9c. Housing and Husbandry	Are the welfare-related assessments and interventions that were carried out prior to, during, or after the experiment reported?			
19.	10a. Sample Size	Is the total number of animals used in each experiment, **AND** the number of animals in each experimental group specified?			
20.	10b. Sample Size	Is the explanation on sample size calculation **OR** justification provided?			
21.	10c. Sample Size	Is the number of independent replications of each experiment indicated?<br><br>(N/A= If a single procedure was performed)			
22.	11a. Allocating Animals	Is the allocation approach to different study groups **OR** randomization **OR** matching mentioned?<br><br>(N/A= If there was a single experimental group)			
23.	11b. Allocating Animals	Is the order in which the animals in the different experimental groups were treated and assessed mentioned?			
24.	12. Experimental outcomes	Are the primary and secondary experimental outcomes assessed (e.g. fistula, stricture, etc.) clearly described?			

25.	13a. Statistical methods	Are details about the statistical methods mentioned?			
26.	13b. Statistical methods	Is the unit of analysis for each dataset mentioned?			
27.	13c. Statistical methods	Are there any methods to assess adequacy of statistical approach utilized?			
28.	14. Baseline data	Are the relevant characteristics and health status of animals reported? (e.g. weight **OR** microbiological status **OR** drug or test naïve)			
29.	15a. Numbers analysed	Are the absolute numbers of animals in each group included in each analysis reported?			
30.	15b. Numbers analysed	Is there an explanation on animals or data that were not included in the study?<br><br>(N/A= If a statement about inclusion of all animals was reported)			
31.	16. Outcomes and estimation	Are the results for each analysis carried out, with a measure of precision (e.g. standard error or confidence interval) reported?			
32.	17a. Adverse events	Are details of all-important adverse events in each experimental group given?<br><br>(N/A= If a statement about absence of adverse events was reported)			
33.	17b. Adverse events	Are modifications to the experimental protocols made to reduce adverse events described?<br><br>(N/A= If a statement about absence of adverse events was reported)			

34.	18a. Interpretation/ Scientific Implications	Are the results interpreted, considering the study objectives and hypotheses, current theory and other relevant studies in the literature?			
35.	18b. Interpretation/ Scientific Implications	Is there any comment on the study limitations including any potential sources of bias, any limitations of the animal model, and the imprecision associated with the results?			
36.	18c. Interpretation/ Scientific Implications	Is there any description on the implications of the experimental methods or findings for the replacement, refinement or reduction (the 3Rs) of the use of animals in research?			
37.	19a. Generalizability/ Translation	Is there any comment on the translatability of the study findings to other species or systems including relevance to human biology?			
38.	20. Fund	Is there a mention on funding sources?			
