# Supplementary material for: Quality of Reporting in Preclinical Urethral Tissue Engineering Studies: A Systematic Review to Assess Adherence to the ARRIVE Guidelines
Source: Animals (Basel). 2021 Aug 21;11(8):2456. doi: 10.3390/ani11082456 (PMC8388767; doi:10.3390/ani11082456)
Supplement: Supplementary file 1 [file animals-11-02456-s001.zip › Table S2.pdf]

Supplementary Table S2.

Summary of the main aspects of the analyzed studies. Abbreviations: BAMG = Bladder acellular matrix graft, PLA = polylactic acid, PLLA/PEG = Poly-L-lactic acid/ Poly(ethylene glycol), SIS = Small intestinal submucosa, P(LA/CL) = Poly(lactide-co-caprolactone), NZW = New Zealand white, NM = Not mentioned, M= Male, F = Female, PLCL/PTMC = Poly(l-lactide-co-ε-caprolactone) and Poly(trimethylene carbonate).

No.	Author	Year	Ref.	Strain	Sex	Age (Months)	Wt. (Kg)	Number of Animals	Graft approach (defect length in mm)	Graft Material	Follow up Duration (months)
1.	Huang J-W	2014	[34]	NZW	M	NM	2	30	Patch (15)	BAM, rabbit	3
2.	Li H	2014	[35]	NZW	M	NM	NM	36	Patch (20)	BAM, rabbit	6
3.	Wang F	2014	[36]	NZW	M	NM	NM	12	Patch (10)	Denuded amniotic scaffold, human	3
4.	Kajbafzadeh A-M	2014	[37]	NM	M	NM	NM	12	Patch (5)	Preputial acellular matrix, human	3
5.	Sun D	2014	[38]	NZW	M	NM	NM	28	Patch (5)	Subcutaneous implanted<br>Human umbilical cord	3

6.	Xu Y	2014	[39]	NZ	M+F	8	NM	21	Patch (5)	Subcutaneous implanted muscle microsomes	1
7.	A Da Silva	2014	[40]	NZW	M	NM	NM	16	Tube (10)	Collagen, porcine	3
8.	Chun SY	2015	[41]	NZW	M	NM	NM	20	Patch (20)	BAM, porcine	6
9.	Guo H	2016	[42]	NZW	M	NM	1.5-2.0	24	Patch (20)	SIS, porcine	6
10.	Huang J-W	2015	[43]	NZW	M	NM	2-2.5	30	Patch (20)	Cellulose, bacterial	3
11.	Zhang K	2015	[44]	NZW	M	NM	NM	12	Patch (20)	P(LA/CL) + collagen (source not mentioned)	0.5
12.	Wang DJ	2015	[45]	NZW	M	3	2-2.5	24	Patch (10)	PLA	1.5
13.	Rogovaya O	2015	[46]	Chinchilia Rabbits	M	NM	2	20	Tube (15)	Keratinocyte living skin equivalent	3
14.	Lv X	2016a	[47]	NZW	M	NM	NM	18	Patch (15)	Keratin/silk fibroin	6
15.	Lv X	2016b	[48]	NZW	M	3	2.5-3	27	Patch (20)	PLLA/PEG	3

16.	Pinnagoda K	2016	[49]	NZW	M	NM	2.5–3.5	20	Tube (20)	Collagen, porcine	9
17.	Gunes M	2016	[50]	NZW	M	2	2.5-3	12	Patch (10)	Buccal Mucosa and Amniotic Membrane, rabbit	2
18.	Nikolavsky D	2016	[51]	NZW	M	Post pubertal	2.8-3.4	16	Patch “liquid” (10)	Liquid Buccal Mucosal Graft, rabbit	6
19.	Yang Liu	2017	[52]	NZW	M	NM	2.0–2.5	24	Patch (20)	SIS, porcine	3
20.	Jiang S	2017	[53]	NZW	M	6-8	2.5–4	18	Tube (15)	Mesothelial Cell-Seeded Autogenous Granulation Tissue	6
21.	Larsson, HM	2018	[54]	NZW	M	NM	2.5–3.5	7	Tube (20)	Collagen, bovine	6
22.	Algarrahi K	2018	[55]	NZW	M	NM	3-3.5	12	Patch (10)	Silk Fibroin	3
23.	Sa, Y	2018	[56]	NZW	M	NM	1.5-2	20	Patch (20)	Bladder acellular matrix graft BAMG, source not mentioned	5
24.	Sartoneva R	2018	[57]	NZW	M	NM	NM	34	Patch (20)	PLCL/PTMC	4

25.	Pusateri CR	2019	[58]	NZW	M	5	3.2 (2.8–3.5)	10	Patch (15)	Decellularized Placental Membrane, human	12
26.	Guo H-L	2019	[59]	NZW	M	NM	2.5-3	30	Tube (15)	prevascularized capsular tissue prelaminated with buccal mucosa, rabbit	12
27.	Hua X	2019	[60]	NZ	M	Adult	2.5-3.0	30	Patch (20)	Tunica Vaginalis, rabbit	6
28.	Li Y	2017	[61]	NZW	M	MN	2-2.5	10	Tube (30)	Collagen, rabbit	2
